# Introducing *qrlabelr*: Fast user-friendly software for machine- and human-readable labels in agricultural research and development

**DOI:** 10.12688/gatesopenres.15268.1

**Published:** 2024-03-27

**Authors:** Alexander Kena, Ebenezer Ogoe, Clara Cruet-Burgos, Richard Agyare, Naomi Adoma, Benjamin Annor, Rubi Raymundo, Geoffrey Morris

**Affiliations:** 1 Soil and Crop Sciences, Colorado State University, Fort Collins, Colorado, 80523, USA; 2 Crop and Soil Sciences, Kwame Nkrumah University of Science and Technology, Kumasi, Ashanti, PMB, Ghana; 3 Council for Scientific and Industrial Research-Savanna Agricultural Research Institute, Nyankpala/Tamale, Northern region, Ghana; 4 Council for Scientific and Industrial Research-Crops Research Institute, Fumesua/Kumasi, Ashanti, Ghana

**Keywords:** Agricultural experiments, plot labels, open-source software, QR codes

## Abstract

The advent of modern tools in agricultural experiments, digital data collection, and high-throughput phenotyping have necessitated field plot labels that are both machine- and human-readable. Such labels are usually made with commercial software, which are often inaccessible to under-funded research programs in developing countries. The availability of free fit-for-purpose label design software to under-funded research programs in developing countries would address one of the main roadblocks to modernizing agricultural research. The goal was to develop a new open-source software with design features well-suited for field trials and other agricultural experiments. We report here *qrlabelr*, a new software for creating print-ready plot labels that builds on the foundation of an existing open-source program. The *qrlabelr* software offers more flexibility in the label design steps, guarantees true string fidelity after QR encoding, and provides faster label generation to users. The new software is available as an R package and offers customizable functions for generating plot labels. For non-R users or beginners in R programming, the package provides an interactive Shiny app version that can be launched from R locally or accessed online at https://bit.ly/3Sud4xy. The design philosophy of this new program emphasizes the adoption of best practices in plot label design to enhance reproducibility, tracking, and accurate data curation in agricultural research and development studies.

## Introduction

The introduction of digital data collection and high-throughput phenotyping tools in modern field trials, has made it imperative to design experimental plot labels that are both machine- and human-readable. A machine- and human-readable plot label, by design, displays meaningful human-legible texts about experimental plots alongside a machine-decodable barcode that stores unique identifiers for each plot ( Copp *et al.*, 2014; Diazgranados & Funk, 2013; Wu *et al.*, 2020a). The capacity to easily create machine-readable plot labels would ensure the safe deployment of these modern tools in field experiments ( Rife & Poland, 2014).

The addition of barcodes to plot labels has been shown to be useful in enhancing the reproducibility of experiments, tracking, and accurate data curation ( Baker, 2016; Copp *et al.*, 2014). Since humans cannot naturally read barcodes, a machine-readable label becomes human-readable when it shows meaningful texts about experimental plots alongside the barcode. It is best practice for each plot label to display alongside a barcode, human-readable texts such as plot number, replication number, plot position or coordinates in a grid layout, entry name or treatment assigned to the plot, and trial location.

To easily generate machine- and human-readable plot labels, agricultural researchers in advanced programs mostly resort to the use of licensed commercial software such as BarTender _®_ ( https://www.seagullscientific.com/) or MS Office. However, resource-limited researchers working at the various National Agricultural Research and Extension Systems (NARES) in the Global South, typically do not have budget lines to acquire these licensed software or may find the process of generating plot labels using these software cumbersome and complicated. Consequently, such programs resort to the use of error-prone and time-consuming hand-written labels, or labels that are not machine-readable. The lack of free and accessible software for creating both machine- and human-readable field plot labels precludes these researchers from benefiting from the deployment of digital data collection and high-throughput phenotyping tools ( Rife & Poland, 2014).

To this end, open-source software provides a viable alternative to licensed commercial software. However, existing open-source software for creating plot labels affixed with barcodes, *baRcodeR* ( Wu *et al.*, 2020b) may not be fit for purpose with respect to designing machine- and human-readable labels for field experiments. The *baRcodeR* package was developed using the R programming language ( R Core Team, 2022) and became available on the Comprehensive R Archive Network (CRAN) as a package in 2018. This open-source software, however, has a design bias toward generating machine-readable labels, and does not allow for the incorporation of additional meaningful human-readable text items about experimental plots and trials.

Among barcodes, the Quick Response (QR) codes offer better features including high data encoding capacity, small printout size, as well as dirt and damage resistance. The *baRcodeR* package provides a QR code generation functionality for label design. However, *baRcodeR* does not support additional text customization, and most importantly, generated QR codes lack true string fidelity when scanned. When strings separated by underscores are submitted to the *baRcodeR* software, it replaces them with hyphens before generating QR codes. This unexpected behavior creates a mismatch and tracking problems during data collection. This is because strings returned from QR code scans would differ from the original strings which had underscores in the researcher’s fieldbook. Another notable deficiency of the *baRcodeR* package with regard to its QR code generation functionality is that it is slow and time intensive as the number of QR codes to generate increases. This undesirable feature stems from the use of the *qrcode* package ( Onkelinx & Teh, 2022) in R that is not C-backed. These deficiencies make the *baRcodeR* software unsuitable for creating plot labels to support research. The mismatching behavior makes its labels incompatible with the popular open-source digital data collection app, Field Book ( Rife & Poland, 2014).

Our goal was to develop a new open-source software for machine- and human-readable plot labels with design features that are well-suited for field trials and experiments and facilitate best practices in breeding operations and data management. We report here the *qrlabelr* package as a new open-source program for creating print-ready plot labels that are both machine- and human-readable. This new program reimagined the *baRcodeR* software and built on its foundation by harnessing new capabilities available in the R programming language. The new software offers flexibility in the label design steps; guarantees true string fidelity after QR encoding; and provides faster plot label generation even though it incorporates more information into the label. The software is available as an R package and offers customizable functions for generating plot labels. For non-R users or beginners in R programming, the package provides an interactive Shiny app version that can be launched from R locally or accessed online.

## Methods

We developed *qrlabelr* as an R package to provide a free and accessible software environment to the agricultural research community in the developing world. R’s modern features support the development and incorporation of interactive web applications that can be run locally on a machine or deployed to a server for online access. R packages are instrumental in organizing, encapsulating, and distributing code, making them an indispensable asset for reproducible research in the R programming ecosystem.

### Package structure

**Figure 1.**
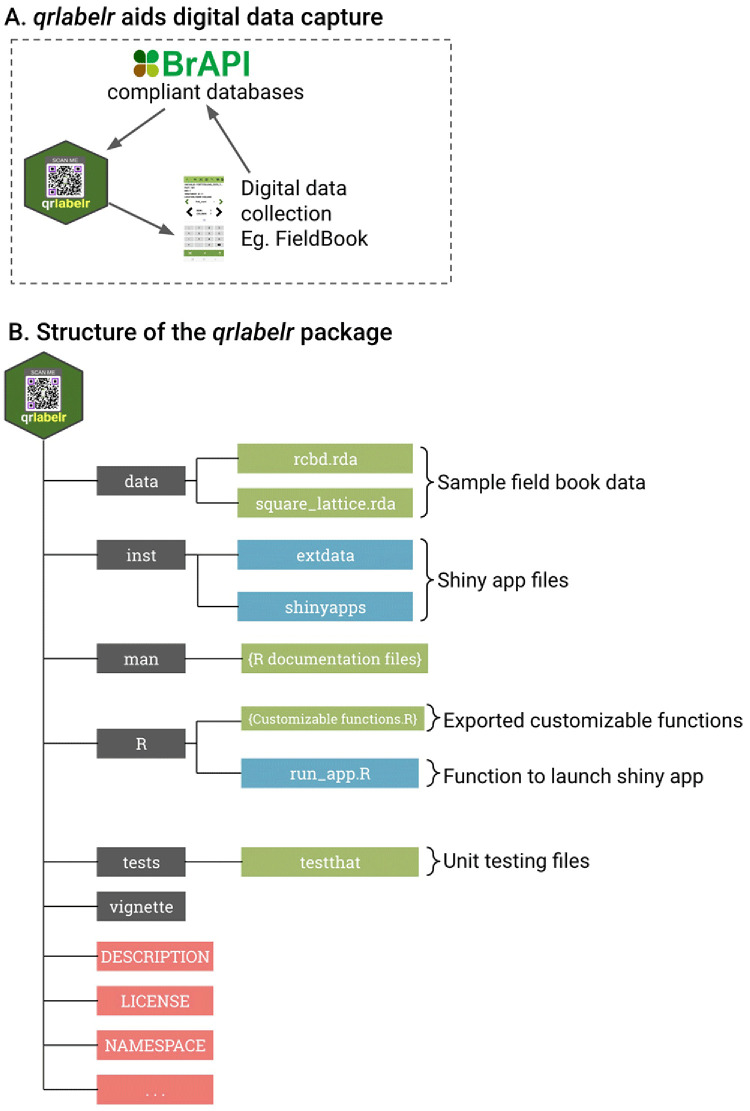
The purpose and structure of the qrlabelr package. ( **A**) The purpose of the *qrlabelr* package is to aid electronic data capture using digital data collection tools such as Field Book. ( **B**) The package provides two main options for label design using either customizable functions in R or a user-friendly Shiny app. The ‘data’ folder contains exported sample field book data based on complete and incomplete block designs. The ‘inst’ folder consists of scripts and supporting files for the user-friendly shiny app. The 'man' folder comprises manual pages for our package functions, which provide detailed documentation for individual functions, including descriptions, usage examples, and arguments. The ‘R’ folder includes scripts for all exported functions for qrlabelr that can be invoked in R. The ‘tests’ folder has scripts for automated unit testing of all functions and data files in qrlabelr via the testthat package. The ‘vignette’ folder contains a detailed user guide for functions and use cases for qrlabelr. The DESCRIPTION file is made up of metadata about the package, such as the package name, version, author information, license, and specification of dependencies on other R packages. The NAMESPACE file defines the package's namespace, specifying which functions and objects are meant to be accessible to users. Folders are highlighted in grey color and other standalone files are highlighted in red. Package components associated with the shiny app are highlighted in light blue, whereas all other files are highlighted in green color.

We describe the key components and directories of the *qrlabelr* package in Figure 1. This structure follows best practices recommended for R package development to ensure efficient organization, reusability, and sharing with the R community.

The *qrlabelr* package comprises core R functions stored in the ‘R’ folder; a 'man' folder for function and exported data documentation; an ‘inst’ folder for files associated with its shiny app; as well as a DESCRIPTION file for package metadata, and an NAMESPACE file to manage function accessibility and encapsulation.

### Package development tools

We used RStudio’s package build tools ( Posit team, 2023) to create, write, organize, and test scripts for *qrlabelr*. The *devtools* (v.2.4.5) ( Wickham *et al.*, 2022b) and *usethis* (v.2.2.2) ( Wickham *et al.*, 2023a) packages facilitated Git and GitHub integration for version control and tracking. We used the *testthat* package (v.3.2.0) ( Wickham *et al.*, 2023e) to write unit tests for all exported functions in *qrlabelr*. We obtained code/test coverage for *qrlabelr* using the *covr* package (v.3.6.3) ( Hester *et al.*, 2023). The *qrlabelr* package has a code coverage of 94% ( https://app.codecov.io/gh/awkena/qrlabelr).

To check the *qrlabelr* compatibility with various operating system (OS) platforms, we used a variety of approaches. First, we used the *devtools::check()* function to perform R-CMD-check. Second, we setup a GitHub Action Workflow to automatically run R-CMD-check on macos-latest (release), windows-latest (release), ubuntu-latest (devel), ubuntu-latest (release), and ubuntu-latest (oldrel-1) after every commit. Third, we performed further checks on Ubuntu Linux 20.04.1 LTS, R-release, GCC, Fedora Linux, R-devel, clang, gfortran, and Windows Server 2022, R-devel, 64 bit using the *check_for_cran()* function in the *rhub* package (v.1.1.2) ( Csárdi *et al.*, 2022).

We specified the dependencies of *qrlabelr* ( Table 1) in the package DESCRIPTION file using the *usethis::use_package()* function. To create the vignette for *qrlabelr*, we used *usethis::use_vignette()* function to setup the metadata for the vignette. The vignette builder for *qrlabelr* is the *knitr* package. Similarly, we used the *usethis::use_readme_rmd() devtools::build_readme()* functions to create the README file for *qrlabelr*.

**Table 1.** Dependencies for the *qrlabelr* package functionality.

Package	Minimum version	Description	Author(s)
argonDash	≥ 0.2.0	Argon Shiny dashboard template	Granjon (2019a)
argonR	≥ 0.2.0	R interface to Argon HTML design	Granjon (2019b)
assertthat	≥ 0.2.1	Easy Pre and Post Assertions	Wickham (2019)
bslib	≥ 0.4.2	Custom 'Bootstrap' 'Sass' Themes for 'shiny' and 'rmarkdown'	Sievert *et al.* (2023)
dplyr	≥ 1.0.10	A fast, consistent tool for working with data frame like objects, both in memory and out of memory	Wickham *et al.* (2023b)
desplot	≥ 1.10	Plotting Field Plans for Agricultural Experiments	Wright (2023)
ggplot2	≥ 3.4.2	Create Elegant Data Visualisations Using the Grammar of Graphics	Wickham *et al.* (2023c)
purrr	≥ 1.0.1	A complete and consistent functional programming toolkit for R	Wickham and Henry (2023)
QBMS	≥ 0.9.1	QBMS: Query the Breeding Management System(s)	Al-Shamaa *et al.* (2023)
qrencoder	≥ 0.1.0	Quick response code (QR Code) / matrix barcode creator	Rudis (2016)
raster	≥ 3.6.23	Geographic data analysis and modeling. Reading, writing, manipulating, analyzing and modeling of spatial data	Hijmans *et al.* (2023)
reactable	≥ 0.4.3	Interactive data tables for R, based on the React Table JavaScript library	Lin *et al.* (2023)
readxl	≥ 1.4.1	Read Excel Files	Wickham *et al.* (2023d)
shiny	≥ 1.7.5.1	Makes it incredibly easy to build interactive web applications with R	Chang *et al.* (2023)
shinyBS	≥ 0.61.1	Twitter bootstrap bomponents for Shiny	Bailey (2022)
shinycssloaders	≥ 1.0.0	Add loading animations to a 'shiny' output while it is recalculating	Sali *et al.* (2020)
shinyjs	≥ 2.1.0	Easily improve the user experience of your Shiny apps in seconds	Attali (2021)
shinyWidgets	≥ 0.7.6	Collection of custom input controls and user interface components for 'Shiny' applications	Perrier *et al.* (2023)
uuid	≥ 1.1-1	Tools for generating and handling of UUIDs	Urbanek and Tso (2022)

To document exported functions and data in *qrlabelr*, we invoked the *devtools::document()* function which converted R *oxygen2* (v.7.2.3) ( Wickham *et al.*, 2022a) comments, examples and tags into documentations.

Having passed all CRAN checks on all OS platforms, we submitted *qrlabelr* to CRAN for release via the *devtools::release()* function.

### Requirements

Running the *qrlabelr* package on a local computer requires specific packages detailed in Table 1. These dependency packages are installed automatically during package installation of *qrlabelr* in R.

### Installation

To install the stable version of *qrlabelr* (v.0.2.0) from CRAN, users need to have R (>= v.4.1.0) and the RStudio IDE installed first. Before installation, R and RStudio, as well as already installed packages, must be up to date. Next, users can install the package via different command lines. First, users can open RStudio and install the package and its dependencies:


> install.packages("qrlabelr", dependencies = TRUE)


Second, users can install the development version of *qrlabelr* from GitHub:


> install.packages("devtools")
> devtools::install_github("awkena/qrlabelr")


Once the installation is complete, users can load the package by entering the following command in the console:


> library(qrlabelr)


## Main features of the *qrlabelr* package

### The *qrlabelr* package is software for both R and non-R users

**Figure 2.**
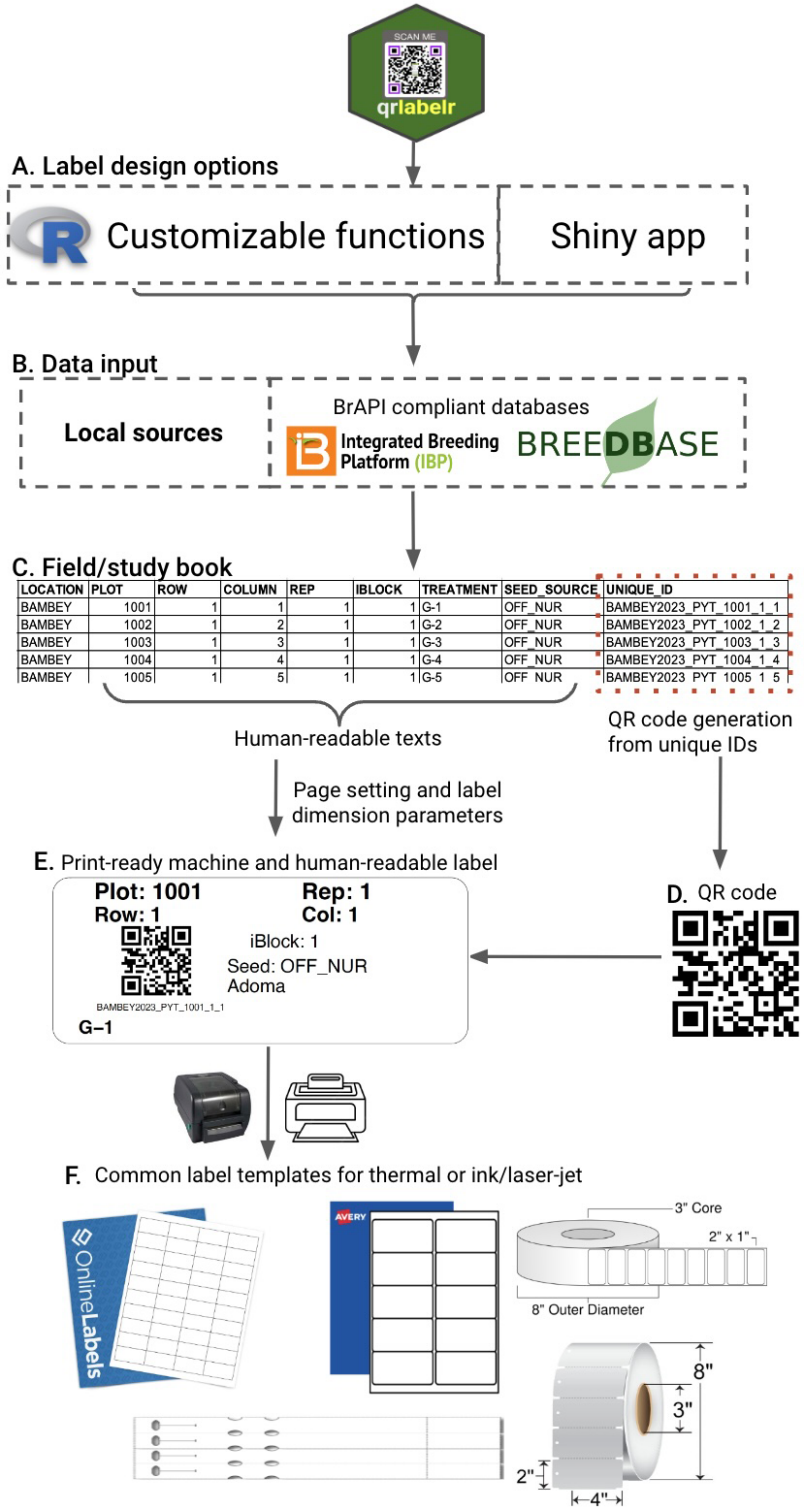
Designing custom print-ready machine- and human-readable plot labels that are compatible with widely used label templates using the *qrlabelr* package. **A**– **C**: The two user-centered options provided by *qrlabelr* both take a field/study book with plot or sample attributes as input data from either local or BrAPI-supported databases; generate QR codes using unique plot or sample IDs ( **D**); and combine the generated QR code with human-readable texts to design machine- and human-readable labels ( **E**). The program accepts custom page and label dimension parameters to make the labels print-ready on common label templates for thermal or ink/laser-jet printing ( **F**).

The *qrlabelr* package offers two user-centered options for creating labels affixed with QR codes ( Figure 2A). The app is user-friendly, allowing users to create accurate and highly informative plot labels similar to those generated by commercial software.

The first option provides a helper function to access an interactive Shiny app (EasyQrlabelr) for R-beginners or non-R users who may find working in R uncomfortable. This option facilitates users to run the Shiny app using their computer as a host without an internet connection. The second option involves the use of customizable functions to create rectangular field plot labels or any rectangular general-purpose labels embossed with QR codes. This option is for R users who can automatize these functions.

Both the Shiny app and customizable functions deliver the exact same features, so it all comes down to a user's preference.

### The *qrlabelr* package uses a multi-column field or study book as input data

To use *qrlabelr*, one must first generate a field or study book that shows plot or sample attributes ( Figure 2C). Typically, we create plot attributes in the field book based on the experimental design and treatment randomization. Following best practices, we recommend the inclusion of the grid coordinates of plots (row and column/range numbers of plots) in the field book for field plot labels.

There are free open-source softwares such as the *FielDHub* package ( Murillo *et al.*, 2021), which users can use to easily generate an input field book for plot label design in *qrlabelr*. Other user-preferred software such as the breeding management system (BMS) can equally be used to generate an input field book if desired.

In R, import the input field book as a data frame. In the Shiny app, suitable file formats for import into the application are standard UTF-8 encoded comma delimited or separated values (.csv) or MS-Excel (.xls/.xlsx) files. There are no restrictions on the order of columns or column names in the input data. We, however, recommend the use of concise and meaningful column names to easily identify the information they carry ( Figure 2C).

### The *qrlabelr* package is BrAPI-compliant

Users can import input data directly from BrAPI-supported breeding databases such as BMS and Breedbase into *qrlabelr* in R or using the shiny app ( Figure 2B). This interoperability functionality, via the *QBMS* package, enhances flexibility in data import into *qrlabelr* and allows for seamless communication between *qrlabelr* and breeding databases. This eliminates the need to download trial or study books designed for experiments or seed sample lists from these databases before importing from local sources into *qrlabelr*.

### The *qrlabelr* package designs print-ready machine- and human-readable labels

**Figure 3.**
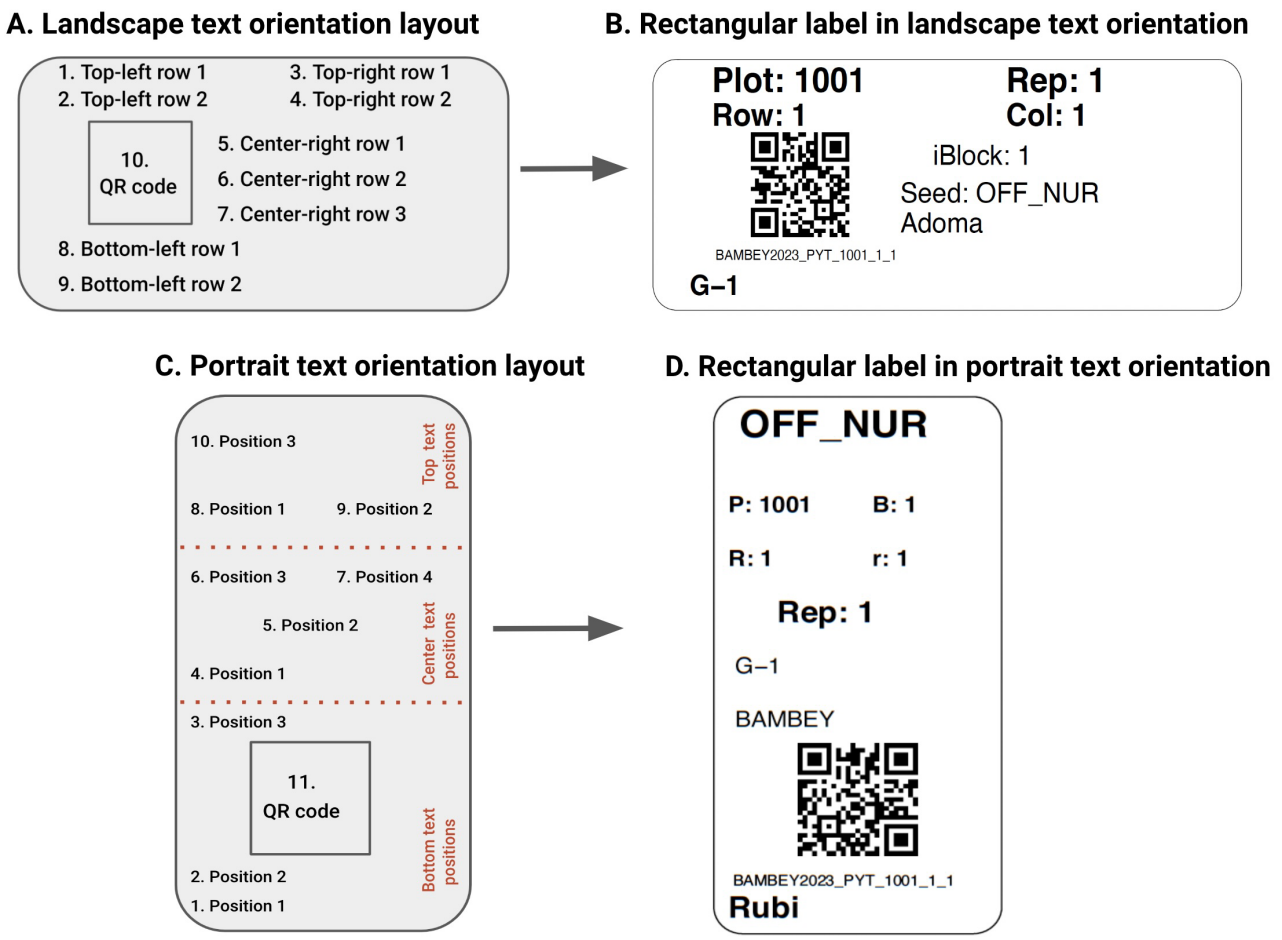
Text orientation formats in *qrlabelr*. **A**: Landscape text orientation format showing nine (9) delineated text positions and 1 QR code position for any rectangular label. **B**: An example of a rectangular label designed using the landscape text orientation format. **C**: Portrait text orientation format showing ten (10) delineated text positions and 1 QR code position for any rectangular label. **D**: An example of a rectangular label designed using the portrait text orientation format in *qrlabelr*. The choice of either a portrait or landscape text orientation format is based on the user’s preference.

The software allows users to design rectangular labels that display both human-readable texts and a machine-readable QR code ( Figure 2D-E). The program uses designated unique identifiers for each plot or sample to generate QR codes ( Figure 2D). The type of label to design determines the specific human-readable texts to show on the label ( Figure 3). The available label types are field plot and general-purpose labels.

The field plot label option is available through the ***field_label()*** function in R, or the **‘Field plot label**’ in the Shiny app. In R, the general-purpose label option is available via the ***gp_label()*** and the ***gp_label_portrait()*** customizable functions. In the Shiny app, users can access the general-purpose label option through the **‘General-purpose landscape text label’** and the **‘General-purpose portrait text label’** options. 

Generally, the user can pass any text under any of the columns contained in the input data to the program for onward display on the label. This flexibility makes *qrlabelr* more user-friendly with generated labels showing more meaningful information.

PDF files are the main output files from *qrlabelr*. The program uses page and label dimension settings that are compatible with widely used label templates supplied by popular vendors to generate the PDF files ( Figure 2F). Users can, thus, design any rectangular label by passing custom page and label dimension parameters to the program. The program saves the generated PDF file automatically to the working directory in R. In the Shiny app, users can download the PDF output using download buttons.

### The *qrlabelr* package provides landscape and portrait text orientation formats

The *qrlabelr* package can design rectangular labels in a landscape or portrait text orientation ( Figure 3). The landscape text orientation format comes with nine (9) delineated text positions in addition to the QR code position ( Figure 3A). In the portrait text orientation format, ten (10) delineated text positions are available to users ( Figure 3B). Users can fill out these delineated text positions with human-readable texts based on their preference.

For a field plot label option, the program uses a landscape text orientation by default. Figure 3A shows how the program maps the nine (9) text positions by default to the fixed human-readable texts on the label. The two top-left row positions map to Plot ID and Row ID. The two top-right row positions map to Rep ID and Column/Range ID. The program maps the three center-right row positions to the intra-block ID number if present, seed source for entries (optional) and name of researcher (optional). Finally, the two bottom-left row positions map to the Location of experiment or trial and Entry/Treatment name, respectively.

Users can, however, override these default mappings for field plot labels with their preferred texts via the general-purpose label option.

### The *qrlabelr* package implements a faster C-compiled QR code generation library

Affixing QR codes on plot labels makes them machine-readable for easy plot identification and tracking. However, generating QR codes from strings could be computationally intensive depending on the number and length of strings to encode into QR codes, and the error correction level for the QR codes.

**Figure 4.**
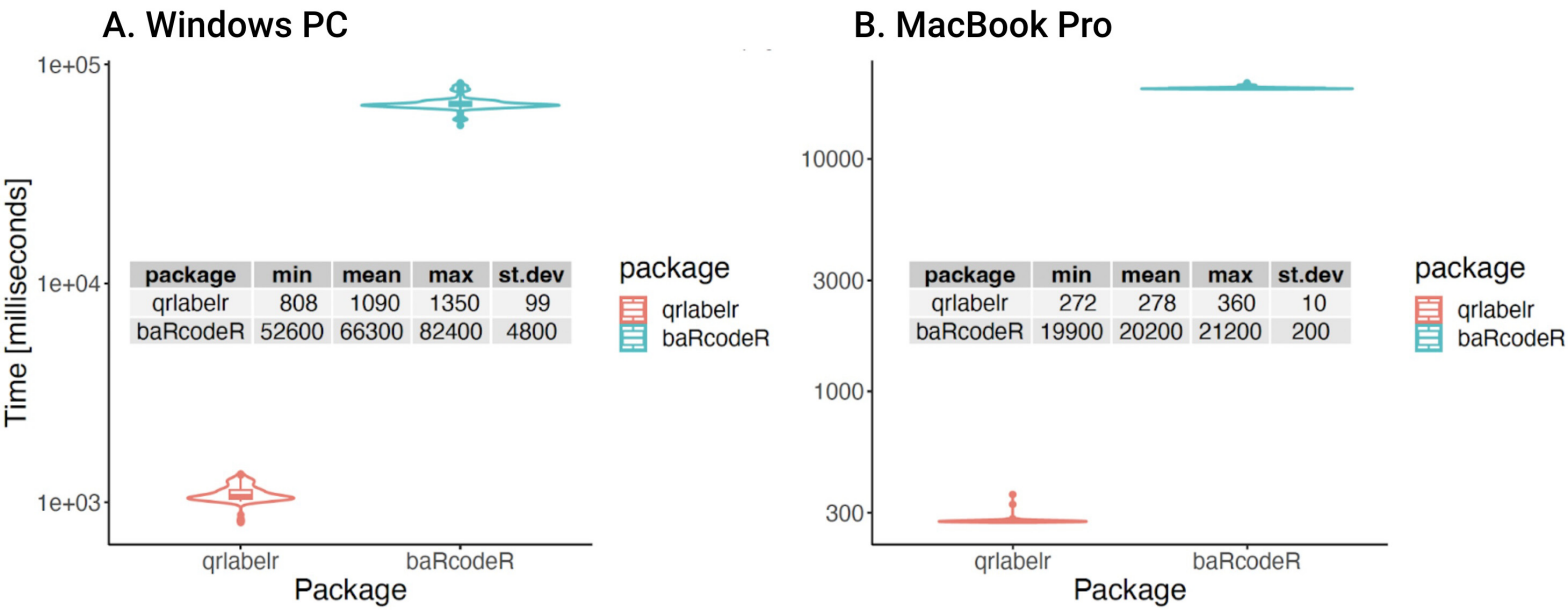
Microbenchmarking the QR code generation functions in *qrlabelr* and *baRcodeR* packages. The microbenchmarking measured the time used by the two packages to generate 200 QR codes on a Windows PC ( **A**) and a MacBook Pro ( **B**). The Windows PC had 8 cores; Processor: Intel(R) Core(TM) i7-10510U CPU @ 1.80GHz 2.30 GHz; Installed RAM: 16.0 GB (15.7 GB usable); System type: 64-bit operating system, x64-based. The MacBook Pro had 12 cores; Chip: Apple M2 Max; Memory: 32 GB; macOS: Sonoma 14.2; Platform: aarch64-apple-darwin20 (64-bit). Table inset is the summary statistics for the two packages for 100 microbenchmarking runs measured in milliseconds (rounded to three significant figures). The results show that the QR code generation function in *qrlabelr* is at least 60 times faster than the equivalent function in the *baRcodeR* package (t-statistic = -136.11; df = 99.1; p value < 2.2e-16 on the Windows PC; and t-statistic = -858.14; df = 99.4; p value < 2.2e-16 on the MacBook Pro).

To generate labels embossed with QR codes faster in R, we implemented a C-compiled and backed library for QR code generation in *qrlabelr* ( Rudis, 2016). The helper function, ***make_qrcode()*** guarantees faster QR code generation when we micro-benchmarked it against an equivalent function in the *baRcodeR* package ( Figure 4). Using the *microbenchmark* package ( Mersmann *et al.*, 2023) in R, we submitted 200 unique plot IDs to the QR code generating functions in both packages and measured the average time it took to complete QR code generation for 100 independent runs. To test the hypothesis that the QR code generation functionality in the qrlabelr package is faster than its equivalent in the baRcodeR package, we used two computers with different processor speed. Figure 4A shows the microbenchmarking results for the two packages on a 64-bit operating system, x64-based Windows HP PC with 8 cores, an Intel(R) Core(TM) i7-10510U CPU @ 1.80GHz 2.30 GHz processor; and an installed RAM of 16.0 GB (15.7 GB usable). Similarly, Figure 4B shows the microbenchmarking results for the two packages on a aarch64-apple-darwin20 (64-bit) MacBook Pro computer with 12 cores; Apple M2 Max chip; and a 32 GB memory; running under the Sonoma 14.2 MacOS. Our results indicate that the ***make_qrcode()*** function in *qrlabelr* is at least 60 times faster than its equivalent function in the *baRcodeR* package ( Figure 4A and B). 

### The *qrlabelr* package supports best practices for generating unique plot IDs

The string for generating QR codes must be unique for each plot or sample, and possibly informative. For field plot label design, we implemented three methods for passing unique IDs for each plot to the *make_qrcode()* function in *qrlabelr*. These methods are **reproducible unique IDs** (RUID), **universal unique IDs** (UUID), and **custom unique IDs** (custom).

**Figure 5.**
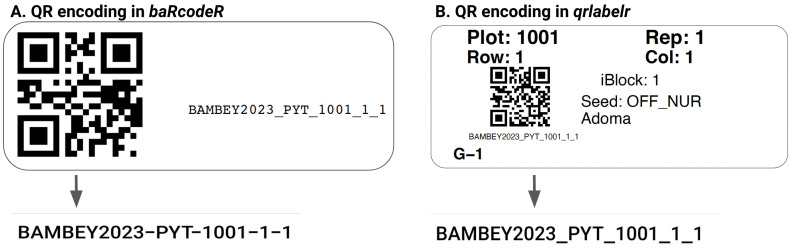
Fidelity of string encoding into QR codes in *baRcodeR* versus *qrlabelr* packages. **A**. QR encoding in *baRcodeR* replaces underscores in the string with hyphens, causing mismatching problems during digital data collection. The *qrlabelr* package guarantees true string fidelity after QR encoding ( **B**).

RUIDs are informative and can be regenerated when provided with the same input field book. For field experiments or trials, we recommend the use of RUIDs. In *qrlabelr*, we generate RUIDs by concatenating LOCATION and year of the experiment, trial name, PLOT, ROW and COLUMN/RANGE IDs for each experimental plot ( Figure 5). For instance, the RUID **BAMBEY2023_PYT_1001_1_1**, represents a unique ID for a plot in location Bambey, in year 2023, in a preliminary yield trial (PYT) and plot number of 1001 with the grid coordinates Row 1 and Column 1.

The UUID method, if selected, produces random time-based unique IDs that are not reproducible and informative, but are highly unique due to their pseudo-random nature.

Users can define their own unique IDs and pass them to the program to generate QR codes if the custom unique ID method is selected. The custom unique ID method must only be used when the input field book contains a column representing unique IDs suitable for QR code generation, as shown in Figure 2C.

Selecting either the RUID or UUID method triggers the program to update the input field book by appending the RUIDs or UUIDs generated to the input field book. The program then automatically saves the updated field book to the working directory in R. A download button for updated field book is available in the Shiny app.

Users can set the desired error correction level (ecl) for generating QR codes. The ecl indicates how much of the QR code is used up for error correction. There are four levels, with 0 (7%) being the lowest level and 3 (30%) being the highest value. For field experiments, we recommend setting the error correction level to 3 (30%) to make them dirt and damage- resistant.

### The *qrlabelr* package guarantees true string fidelity during QR code generation

To prevent mismatching and tracking problems on the field during data collection, the fidelity of the strings encoded into QR codes must not be compromised. The characters in the strings used for QR encoding must be the same characters returned when one scans the QR code. To demonstrate this, we generated QR codes with the string ‘BAMBEY2023_PYT_1001_1_1’ utilizing the existing baRcodeR package and our newly developed *qrlabelr* package ( Figure 5). The *baRcodeR* package replaced the underscores with hyphens, resulting in a modified string: ‘BAMBEY2023-PYT-1001-1-1’ ( Figure 5A). Our program, on the other hand, maintained true string fidelity after QR encoding ( Figure 5B).

### The *qrlabelr* package includes rich documentation and helpful examples

Adequate documentation is a cornerstone of R package development. It serves the crucial purpose of explaining the package's functions, classes, and data structures, making it comprehensible and user-friendly. This section delves into the documentation and examples aspect of our R package development process.

### Documentation Practices

As mentioned earlier, we employed *Roxygen2*, a widely-used documentation system in the R community, to create clear and consistent documentation for our functions. *Roxygen2* is integrated seamlessly with RStudio, streamlining the documentation process. Each function in the package was meticulously documented, including a title, description, usage, arguments, and details regarding the function's output. This ensured that users could easily understand the purpose and functionality of every function. We adhered to a standardized format for documenting functions to promote uniformity and ease of use for both new and experienced R users.

### Examples and Use Cases

In addition to documentation, we provided practical examples and use cases for each function in our package. These examples were carefully crafted to illustrate how to use the functions effectively for various real-world scenarios. Use cases ranged from basic examples for beginners to more advanced scenarios for experienced users. This approach ensured that users of different skill levels could benefit from our package. Our examples included sample input data, function calls, and expected output. This approach facilitates user understanding and encourages the adoption and exploration of our package's capabilities.

### Vignettes

For a more in-depth understanding of complex procedures or workflows within our package, we created vignettes. Vignettes are comprehensive documents that provide step-by-step guidance, along with real data and results. These vignettes covered specific topics or tasks, guiding users through intricate analyses and showcasing the power and versatility of the various functions in our package. Our commitment to comprehensive documentation, practical examples, and user engagement ensures that our R package is approachable and valuable to a diverse user base. By providing clear, well-documented functions and illustrative examples, we empower users to effectively harness the capabilities of the functions in our package.

## Operation

### Launch the Shiny app (EasyQrlabelr) to access a user-friendly GUI of *qrlabelr*

To create rectangular labels using the Shiny app, a user must launch the application either from R or the web. Running the application locally from R is recommended, as it cushions users from unstable internet connectivity. To launch the application from R in your default browser, run this code in the R console:


> qrlabelr::run_app()


**Figure 6.**
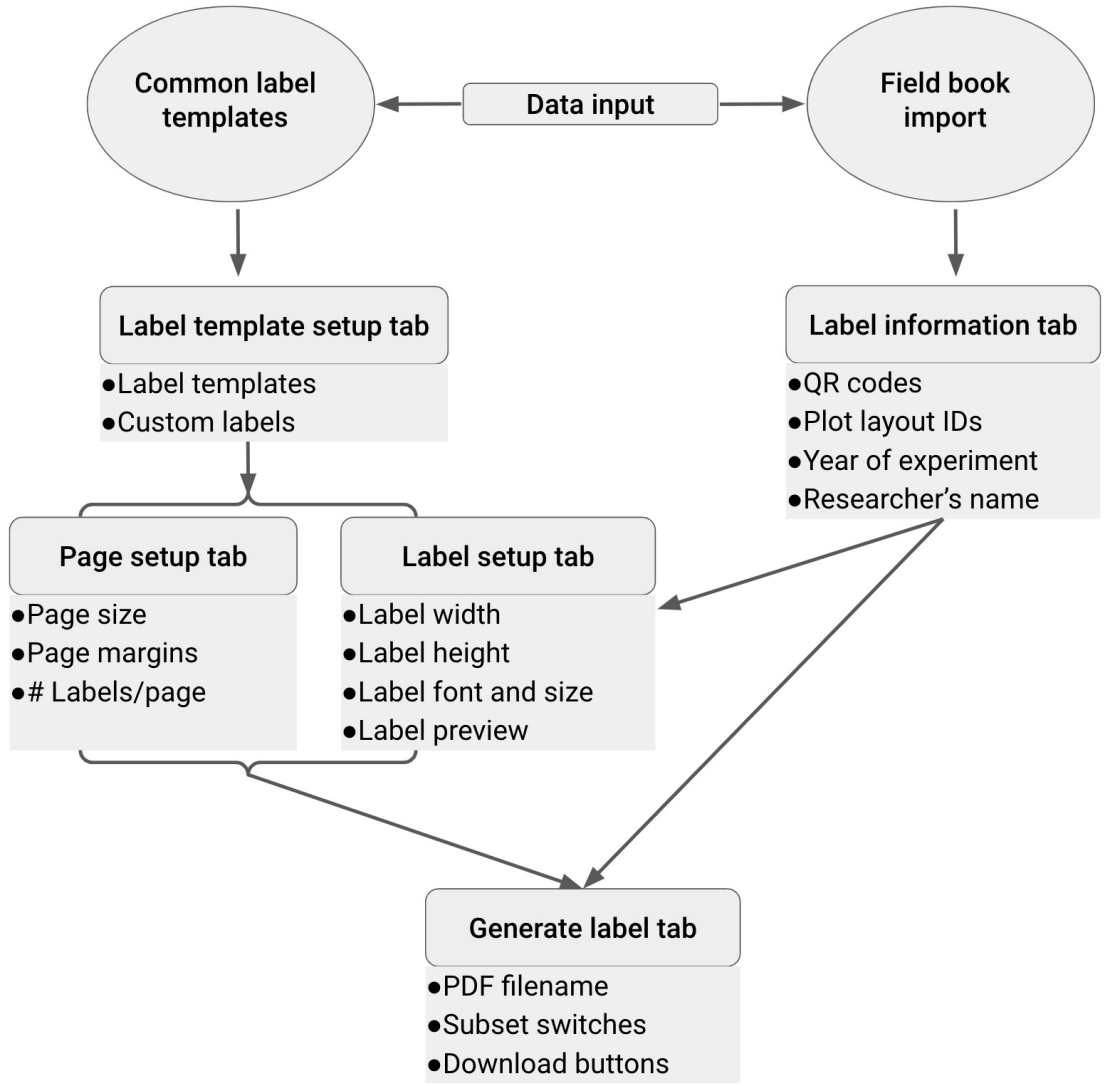
A schematic of the sequential flow of information and steps for creating machine and human-readable labels using the Shiny app in *qrlabelr*. On launching the Shiny app, the program automatically imports a table of common label templates comprising 13 preset templates for designing labels. This imported table is displayed in the Template setup tab and allows the program to determine the page and label setup information when a user selects any of the 13 predefined templates. The app requires users to import a field/study book into the program to be used as input data for QR code generation and human-readable texts in the Label information tab. The app then passes user-defined parameters in the Label information, Page setup and Label setup tabs to the Generate label tab to derive labels as a PDF file for download. Circles represent in-built or user imported data inputs, rectangles represent app tabs and their main functionalities.

This command line opens a new window in your default web browser that shows the EasyQrlabelr Shiny app. The Welcome page provides an overview of the Shiny app, and some quick instructions to get started. A description of the main pages or tabs available in the Shiny app, corresponding features, and the sequential flow of information required to design any rectangular label are depicted in Figure 6.

To successfully generate labels, a user must provide the required inputs by moving from the **Import fieldbook tab** → **Label information tab** → **Template setup tab** → **Page setup tab** → **Label setup and preview tab** → **Generate labels tab.**

The Shiny app offers a wide range of customization options for designing labels. Users can choose from a variety of different templates, label type, and font styles to create labels that meet their specific needs and preferences.

### Invoke customizable functions to create labels in R

Four customizable functions are available in R to create labels ( Figure 1). These functions are the ***create_label(), field_label(), gp_label(),*** and the ***gp_label_portrait().*** The *create_label()* function is the main function for creating labels with a landscape text orientation format, and it is invoked in the *field_label()*, and *gp_label()* wrapper functions. The *gp_label_portrait()* function is a standalone function for creating any general-purpose label in a portrait text orientation.

### Use the customizable *field_label()* function to create field plot labels in R

The *field_label()* function creates rectangular field plot labels in a landscape text orientation based on a label template. It takes arguments for creating field plot labels and passes them to the *create_label()* function. Users can define the page setting and label dimension parameters by using specific arguments for the parameters.

For instance, the code snippet below shows how users can use the the *field_label()* function based on the Avery 94241 template:


> library(qrlabelr)
> field_label(
  dat = qrlabelr::square_lattice,
  wdt = 5, 
  hgt = 2,
  page_wdt = 8.5, 
  page_hgt = 11,
  top_mar = 0.75, 
  bot_mar = 0.75, 
  left_mar = 1.75, 
  right_mar = 1.75, 
  numrow = 4L, 
  numcol = 1L, 
  filename = "mylabel", 
  font_sz = 20, 
  Trial = "PYT", 
  Year = 2023, 
  family = "sans", 
  rounded = TRUE, 
  IBlock = TRUE,
  get_unique_id = "ruid", 
  rname = "AW Kena", 
  seed_source = TRUE, 
  seed_source_id = "SEED_SOURCE",
  ec_level = 3)


To see a detailed documentation for the *field_label()* function in RStudio, run the following codes in the R console:


> ?qrlabelr::field_label


### Use the customizable *gp_label()* and *gp_label_portrait()* functions to create general-purpose labels in R

The *gp_label()* and the *gp_label_portrait()* functions allow for specific user-defined or preferred human-readable texts on a label. These functions give a lot of control and flexibility to users with respect to what human-readable texts, their position, and orientation on the label.

To create any general-purpose label based on the Avery 94220 template with a landscape text orientation, invoke the *gp_label()* function as shown in the code snippet below:


> library(qrlabelr)
> dat <- qrlabelr::square_lattice # Input field book
# Add unique IDs for each plot to input field book
> dat$ids <- paste0(dat$LOCATION,'2023', '_PYT', '_', dat$PLOT, '_', dat$ROW, '_', dat$COLUMN)
> gp_label(
  dat = dat,
  wdt = 2,
  hgt = 1, 
  page_wdt = 8.5, 
  page_hgt = 11,
  top_mar = 0.625,
  bot_mar = 0.625,
  left_mar = 0.625,
  right_mar = 0.625,
  numrow = 8L,
  numcol = 3L,
  filename = 'PlotLabel',
  get_unique_id = 'custom',
  unique_id = 'ids',
  font_sz = 10,
  family = 'sans',
  top_left_txt1 = 'Plot:',
  top_left_txt2 = 'Row:', 
  top_right_txt1 = 'Rep:',
  top_right_txt2 = 'Col:',
  center_right_txt1 = 'iBlock:',
  center_right_txt2 = 'Seed:',
  center_right_txt3 = 'Adoma',
  top_left_id1 = 'PLOT',
  top_left_id2 = 'ROW',
  top_right_id1 = 'REP',
  top_right_id2 = 'COLUMN',
  center_right_id1 = 'IBLOCK',
  center_right_id2 = 'SEED_SOURCE',
  bottom_left_id1 = 'ids',
  bottom_left_id2 = 'TREATMENT',
  ec_level = 1)


The above arguments are passed to the create_label() function to generate the desired labels based on the defined page setting and label dimension parameters.

The code snippet below demonstrates how to use the *gp_label_portrait()* function in R.


> library(qrlabelr)
> dat <- qrlabelr::square_lattice # Input field book
# Add unique IDs for each plot to input field book
> dat$ids <- paste0(dat$LOCATION,'2023', '_PYT', '_', dat$PLOT, '_', dat$ROW, '_', dat$COLUMN)
> gp_label_portrait(
  dat = dat,
  wdt = 2,
  hgt = 1, 
  page_wdt = 8.5, 
  page_hgt = 11,
  top_mar = 0.625,
  bot_mar = 0.625,
  left_mar = 0.625,
  right_mar = 0.625,
  numrow = 8L,
  numcol = 3L,
  filename = 'PlotLabel',
  font_sz = 10,
  family = 'sans', 
  rounded = TRUE,
  bot_txt1 = 'Rubi', 
  cent_txt2 = 'Rep:',  
  cent_txt3 = 'R:', 
  cent_txt4 = 'r:', 
  top_txt1 = 'P:', 
  top_txt2 = 'B:',
  bot_txt2_id = 'ids',
  bot_txt3_id = 'LOCATION',
  cent_txt1_id = 'TREATMENT', 
  cent_txt2_id = 'REP', 
  cent_txt3_id = 'COLUMN', 
  cent_txt4_id = 'ROW', 
  top_txt1_id = 'PLOT',
  top_txt2_id = 'IBLOCK',
  top_txt3_id = 'SEED_SOURCE',
  unique_id = 'ids',
  ec_level = 1)


A detailed documentation for the *gp_label()* and the *gp_label_portrait()* functions in RStudio can be found by running the following codes in the R console:


> ?qrlabelr::gp_label
> ?qrlabelr::gp_label_portrait


### Examples of real use cases of the *qrlabelr* package

**Figure 7.**
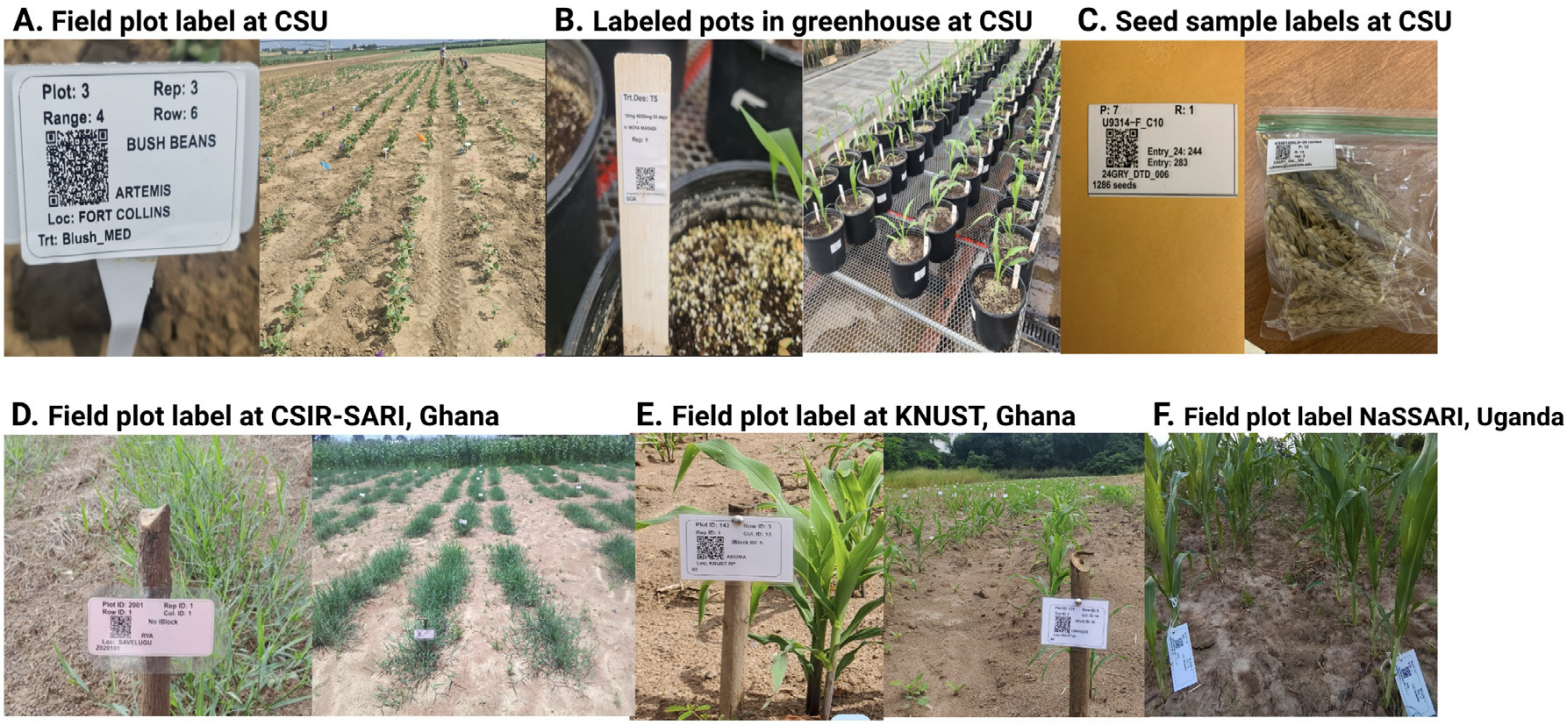
Real use examples of the *qrlabelr* package for designing machine- and human-readable plot and seed sample labels ( **A** – **C**) in the US and in Africa ( **D** – **F**). **A** – **C**: Installed labels from field and greenhouse experiments at Colorado State University (CSU), Fort Collins, USA, printed on weather-proof label paper based on templates by popular vendors; Panels D – F: Installed field plot labels from breeding pipelines fields at the Council for Scientific and Industrial Research-Savannah Agricultural Research Institute (CSIR-SARI), Kwame Nkrumah University of Science and Technology (KNUST), Kumasi, Ghana; National Semi Arid Resources Research Institute (NaSARRI), Uganda. In Panels D – F, the researchers printed plot labels on ordinary A4 sheets using an inkjet printer, and afterwards made the labels waterproof/weatherproof through heat-seal lamination.

Our study followed-up researchers utilizing *qrlabelr* for various applications ( Figure 7). Some cases comprise design labels for field experiments, greenhouse experiments, and seed samples for storage ( Figure 7A–C). These labels were designed using the following templates: Online Label RL2800 template (thermal printing), Online Label OL5125 template (Laser-jet printing), Avery 94237 template, (Laser-jet printing) and the Avery 94207 template (Laser-jet printing).

We collaborated with research programs in Ghana (West Africa) and Uganda, (East Africa) which had no access to weatherproof labels and thermal printers. To address this limitation, we improvised a cost-saving method to get labels printed and weatherproofed ( Figure 7D–E). This improvised method utilized available stationery resources readily at the disposal of these programs. Specifically, researchers utilized our package to print plot labels on ordinary A4 sheets using an inkjet printer, and afterwards made waterproof/weatherproof through heat-seal lamination ( Figure 7D–E). These heat-sealed laminated labels lasted about 4–5 months when used in the field.

## Conclusions

We developed the *qrlabelr* package as an improvement of the *baRcodeR* package to design machine- and human-readable plot labels that are well-suited for field trials. Its enhanced features offer users more flexibility in plot label design options both in R or via the Shiny app. The availability and easy accessibility of the *qrlabelr* package to under-funded research programs in developing countries would address one of the main roadblocks to modernizing agricultural research. The design philosophy of our new program emphasizes the adoption of best practices in field plot label design to enhance reproducibility, tracking, and accurate data curation.

## Ethical considerations

In line with ethical considerations, it is imperative to highlight that we maintain a commitment to user data privacy and security. Our application does not retain or store any user-submitted data for any purpose. Additionally, we do not preserve copies of the generated PDF outputs that contain plot labels. Users are strongly encouraged to download their results onto a suitable medium for offline access and future reference. This commitment to data protection ensures the privacy and confidentiality of user information.

## Data Availability

Microbenchmarking data generated on Windows PC and MacBook Pro computers available on GitHub at https://github.com/awkena/qrlabelr_manuscript. Also available are the scripts for generating the data and plots for Figure 4. The archived source code is available at https://doi.org/10.5281/zenodo.10636681, ( Alexander Wireko Kena, 2024a) and has GNU General Public License, version 3 (GPL-3).

## Software availability

The *qrlabelr* software is currently available as an R package with its source code hosted on GitHub at https://github.com/awkena/qrlabelr. The archived source code can be downloaded from Zenodo at https://doi.org/10.5281/zenodo.10636597 ( Alexander Wireko Kena, 2024b). Users can freely access the package source code, which is available for download or cloning from our GitHub repository using the link above. It is important to note that users are bound by the rights and limitations defined by the GNU General Public License, version 3 (GPL-3). This license ensures that our software remains open and accessible to the community while maintaining the ethical and legal guidelines associated with open-source software distribution.
