# Supplementary material for: Introducing qrlabelr: Fast user-friendly software for machine- and human-readable labels in agricultural research and development
Source: Gates Open Res. 2024 Mar 27;8:28. [Version 1] doi: 10.12688/gatesopenres.15268.1 (PMC11255372; doi:10.12688/gatesopenres.15268.1)
Supplement: Peer review history

# Reviewer report: Reviewer response for version 1

**DOI:** 10.21956/gatesopenres.16616.r36569

**Published:** 2024-07-17

**Authors:** Olivoto Tiago

**Affiliations:** 1 Universidade Federal de Santa Catarina, Florianópolis, State of Santa Catarina, Brazil

The article "Introducing qrlabelr: Fast user-friendly software for machine- and human-readable labels in agricultural research and development" by Alexander Kena et al. presents a new open-source software package, qrlabelr, designed to create print-ready plot labels that are both machine- and human-readable. The qrlabelr package, available in R, offers customizable functions and an interactive Shiny app to facilitate the creation of these labels. The software guarantees true string fidelity after QR encoding and provides faster label generation compared to existing solutions. The article details the software’s development, features, and practical applications in agricultural research.

 The article is well-structured, following a logical flow from the introduction of the problem, through the development and features of the software, to practical applications and conclusions.

 I have a few suggestions to the authors regarding some code bugs I’ve faced while testing the package and how they can be potentially fixed

**get_unique_id argument in gp_label()**

 The function returns an error when get_unique_id is not declared. This probably occurs here https://github.com/awkena/qrlabelr/blob/12003c4c97d1e291f3c8c5329ddf1948eca4151c/R/all_new_functions.R#L1215

 Suggestion

   if (get_unique_id[1] == "uuid") {

     UNIQUE_ID <- uuid::UUIDgenerate(use.time = TRUE, n = nrow(dat), output = "string")

    

   } else if (get_unique_id[1] == "custom") {

     UNIQUE_ID <- dat[, unique_id]

   }

 Set a default value for unique_id

 If I set get_unique_id = “custom”, and I do not have a “unique_id” column, the function returns the following error…This is probably occurs here

https://github.com/awkena/qrlabelr/blob/12003c4c97d1e291f3c8c5329ddf1948eca4151c/R/all_new_functions.R#L323-L328

**Function to Create Unique IDs**

 Add a utility function to create unique IDs if they are not present. This function can concatenate specified columns to generate a unique ID.

 add_unique_id <- function(df, ...) {

     if(!"unique_id" %in% colnames(df)) {

         cols <- as.character(substitute(list(...)))[-1]

         df$unique_id <- apply(df[, cols], 1, function(row) {

             row <- sapply(row, function(x) trimws(as.character(x))) # Convert to character and trim whitespace

             paste(row, collapse = "_")

         })

         return(df)

     } else {

         message("`unique_id` column already exists in the data frame. Skipping...")

     }

 }

**Performance Benchmarking**

 The authors says that “generating QR codes from strings could be computationally intensive depending on the number and length of strings to encode into QR codes, and the error correction level for the QR codes”. So, I would suggest comparing the performance of long (UUIDs) vs shorter unique IDs as well as for the different error correction levels. This should make more robust the comparison of the proposed package with existing solutions.

**Enhanced Plot Generation in add_border Function**

 To produce a field sketch with two locations, I first needed to filter the data for each location. To improve this function, I would suggest first checking if the LOCATION column has more than one level and using a loop to generate plots for each level. This will simplify the generation of multiple field layouts.

     for (loc in unique(dat$LOCATION)) {

 # make the plot here

 ggplot(dat[dat$LOCATION == loc, ], ….)

     }

**Shiny App Improvements**

 The shiny app is well designed and worked well in some tests I’ve made. One suggestion would to add an option in the template setup tab to allow users to "save my own template". This will enhance user experience by enabling the reuse of customized templates.

 By addressing these points, the article will be more scientifically sound and user-friendly, enhancing the overall utility and impact of the qrlabelr package.

Are the conclusions about the tool and its performance adequately supported by the findings presented in the article?

Yes

Is the rationale for developing the new software tool clearly explained?

Yes

Is the description of the software tool technically sound?

Yes

Are sufficient details of the code, methods and analysis (if applicable) provided to allow replication of the software development and its use by others?

Yes

Is sufficient information provided to allow interpretation of the expected output datasets and any results generated using the tool?

Yes

Reviewer Expertise:

Plant breeding, experimental statistics with experience in developing R and shiny apps for R

I confirm that I have read this submission and believe that I have an appropriate level of expertise to confirm that it is of an acceptable scientific standard.

# Reviewer report: Reviewer response for version 1

**DOI:** 10.21956/gatesopenres.16616.r36567

**Published:** 2024-07-17

**Authors:** Jenna Hershberger

**Affiliations:** 1 Clemson University, Clemson, South Carolina, USA

**Summary**

 This manuscript provides a description of a new R package and R Shiny application for agricultural research label creation. The app addresses a need for an open-source method for label generation in plant breeding and genetics research. 

**General comments**

- Why is baRcodeR specifically called out and discussed so deeply throughout the manuscript?  The introduction focuses on the negative aspects of the software to a level that borders on inappropriate, and it is repeatedly referred to throughout the rest of the manuscript in a negative way. Was it created for another use case? Why not contribute to baRcodeR and instead create a new package? Please elaborate on this. Further, baRcodeR is not the only open-source software that can be used for creating plot labels, and Breedbase is one example of another software that can create barcode labels for agricultural research. There are multiple R and python packages/libraries that do this as well. 
- Does this software work for other brands of thermal printers? Do you have a list of compatible hardware?
- "Shiny" is capitalized in some cases and not in others. For example, it is capitalized in the abstract but not in Figure 1. Please make this more consistent.
- The plural for "software" is written as "software" and "softwares" in this manuscript. From a quick search, the correct plural form does not include an s at the end.
- The term field book is also inconsistent throughout the manuscript, written as both one and two words. This is also true in the Shiny app. Please make this consistent.
- BrAPI and the functionality it adds should be introduced in the introduction, as this is a major benefit of this package.
- I recommend moving Table 1 to supplementary files. It would be more useful for users to have a table of functions from the package in the main text rather than the dependencies, especially considering they are automatically installed when the user installs qrlabelr.
- The labels produced by this package have a wide variety of font sizes and alignment within the same label. Please consider standardizing this more for improved human readability.
- This package makes many assumptions about what breeding programs need, but many of the hard-coded assumptions do not work across crops or programs. 
- The language in the manuscript has tone that resembles a sales pitch at times. The "Vignettes" section is a strong example of this tone. Please consider adding more details on the vignettes themselves rather than declaring the value of the vignettes in this section.
- Why does the Shiny app have a different name than the R package? This could add confusion for users, especially given the inconsistent capitalization of the names. The url for the app encoded in the short url in the abstract includes "easyplotlabelr," which is not the name of the title of the page, "EasyQrlabelr," adding additional confusion.
- The website title on the Shiny app includes dark text on a dark background. Consider changing this to increase accessibility.
- The code snippets in the "Operation" section may be a better fit for a supplementary document, maybe an R script with the examples.

 

**Specific comments**

 Abstract

- Multiple comparisons are mentioned ("more flexibility" and "faster"), but it is not clear what qrlabelr is being compared to.
- Consider removing the link in the abstract, changing it to a full link, or at least including the name of the software rather than random numbers and letters in the shortened URL. Using a URL shortener creates a dependency on this third party to maintain your link, which may add risk of link rot. 

Introduction

- There is an unnecessary comma in the first sentence.
- The last sentence in the second paragraph is awkwardly worded. Consider revising.
- In the first sentence of the third paragraph, please consider replacing "in advanced programs" with something more neutral such as "using barcodes." This sentence also includes BarTender with a link but does not spell out Microsoft. Please make references to software consistent in terms of names/links.
- The second sentence of the third paragraph does not need a comma after South.
- In the fifth paragraph, please describe how QR codes provide "dirt and damage resistance."
- In the last paragraph in the introduction, the sentence that starts with "The new software offers" should have commas in place of the semicolons.

Methods

- The second paragraph under the "Package structure" heading has a mix of semicolons and commas. Please revise for consistency.
- In the second paragraph under the "Package development tools" header, "GitHub Action Workflow" should be "GitHub Actions Workflow."
- In the third paragraph under the "Package development tools" header, the word "setup" is a noun only and should be split into two words because it is not used as a noun here.
- The last word of the fourth paragraph under the "Package development tools" header should be singular.
- Under the "Installation" heading, why is RStudio required for installation? Can the package not be run solely on the command line or in a Jupyter notebook? 
- Please clarify the installation instructions to make it clear that these are two different installation options rather than two sequential steps. As written, this is not clear.

Main features of the qrlabelr package

- In the second paragraph, the sentence that starts with "This option facilitates" is awkwardly worded. Please revise.
- What is meant by the term "customizable functions?" Does this just mean that the user can adjust the output using parameters? Or can users change the functions themselves?
- "Automate" may be more appropriate than "automatize," as it is used to refer to software rather than personal behavior.
- In the paragraph following the "The qrlabelr package is BrAPI-compliant" heading, BMS and Breedbase are hyperlinks, but elsewhere urls are written out. Please make this more consistent. The phrase "interoperability functionality" is awkward and should be reworded.
- Under the heading "The qrlabelr package designs print-ready machine- and human-readable labels," the sentence that starts with "The type of label to design" is awkwardly worded. Please revise for clarity.
- In the paragraph beginning with "Generally, the use can," the phrases "more user-friendly" and "more meaningful information" are used, but the package is not compared with anything else. More user-friendly than what?
- Under the heading that starts with "The qrlabelr package provides landscape and portrait…," why is the default field plot label landscape? Please describe this reasoning. "iBlock" is not a common term in plant breeding. Incomplete blocks are often labeled as blocks on field tags. Why not include "Block" rather than "iBlock" here to have the default more aligned with a larger number of field designs?
- In the second paragraph under the heading that starts with "The qrlabelr package implements a faster C-compiled…," there is a citation in the first sentence. Please name the library being cited. The next sentence has an unnecessary comma. Later in this paragraph, the word "speed" should be plural in the sentence ending with "different processor speed." 
- In the paragraph following the header "The qrlabelr package supports best practices…," the phrase "and possibly informative" is unnecessary in the first sentence.
- Please capitalize the acronym "ECL." What does the percentage in parentheses following the ECL levels indicate? It may be useful to add more information on error correction for the reader to help explain how this increases damage resistance.

Operation

- In the second paragraph under the heading "Examples of real use cases of the qrlabelr package," the term "followed-up" does not make sense. Do you mean "followed up with" or "followed?" Additionally, collaborations with research programs in Ghana are mentioned, but the program is not listed in the acknowledgments despite the Ugandan program being mentioned. The comma in this sentence is not in the right place.

Software availability

- This package is on CRAN, but its presence there is not included in this software availability statement.

References

- The reference for the readxl R package includes specific author contributions, which is inconsistent with the other references. Please correct this.
- Most R packages contain specific citation recommendations, but these are not used in the reference section for this manuscript. Please double check all R package references.

Figure 1

- Consider adding the Field Book logo rather than a screenshot for consistency. The Field Book app name is two words, but it is shown as "FieldBook" in this figure.

Figure 3

- The use of numbers in this figure is very confusing. For example, in Figure 3C, "10. Position 3" has mixed numbers. Why give two separate number labels to the same location? Please simplify this.
- In Figure 3B, why are fields 5, 6, and 7 not left aligned together? 
- In general, the wide number of font sizes is very distracting on these labels.
- Why are there only nine fields in the landscape orientation but 10 in the portrait orientation? This does not seem to be described in the text.

Figure 4

- Please standardize the numbers on the y-axis, either using scientific notation on both or neither. Consider using a faceted plot created by ggplot2::facet_grid() or ggplot2::facet_wrap() so the scales match between the two subplots.

Figure 6

- This figure is not easy to follow. Please revise for clarity. The caption mentions 13 template labels, but these are not described in this manuscript. Consider adding this description in supplemental table format.

Are the conclusions about the tool and its performance adequately supported by the findings presented in the article?

Yes

Is the rationale for developing the new software tool clearly explained?

Yes

Is the description of the software tool technically sound?

Yes

Are sufficient details of the code, methods and analysis (if applicable) provided to allow replication of the software development and its use by others?

Yes

Is sufficient information provided to allow interpretation of the expected output datasets and any results generated using the tool?

Yes

Reviewer Expertise:

Plant breeding and genetics, R package development

I confirm that I have read this submission and believe that I have an appropriate level of expertise to confirm that it is of an acceptable scientific standard, however I have significant reservations, as outlined above.

# Reviewer report: Reviewer response for version 1

**DOI:** 10.21956/gatesopenres.16616.r36564

**Published:** 2024-07-17

**Authors:** Adrian Correndo

**Affiliations:** 1 Department of Plant Agriculture, University of Guelph, Guelph, Canada

The article is overall well written, with a clear statement of need, a comparison to the most relevant alternative option in R, description of structure and functionality. Moreover, I really liked how the authors clearly documented the process to create the different package-components, test it, host it on GitHub, and added continuous integration.

 The package looks like a good tool, Congratulations! I would try to implement it myself and recommend others for field experiments labelling.

 Below authors may find some feedback. I hope it helps to polish the article.

 1.  The "Main features" section, which I think it would be better if its subsections are numerated. Also, I strongly advise to remove the first words saying "The qrlabelr package...". That looks like an advertisement. This text could be perfectly removed and will not affect the meaning of subtitles.

 2. I suggest to remove Figure 4. I think this figure and related text in the article as they are not essential content. If it were a machine learning package where the time and computing capacity for running algorithms are more crucial...but not for a package that simply creates labels. I don't think anybody would be too concern about it.

 3. Operation section. I think it would really benefit from adding numbers to the subtitles (i.e. 1-Shiny-App, 2- Create labels in R)

 4. Shiny-App. I couldn't find within the article text a link to the web-based "EasyQrlabelr". Could you please add it? Many users may prefer run it on the web rather than local, so it would be a good option.

 5. I may also suggest adding a screenshot of the EasyQrlabelr shiny-app. I think that would be better than even Figure 6.

Are the conclusions about the tool and its performance adequately supported by the findings presented in the article?

Yes

Is the rationale for developing the new software tool clearly explained?

Yes

Is the description of the software tool technically sound?

Yes

Are sufficient details of the code, methods and analysis (if applicable) provided to allow replication of the software development and its use by others?

Yes

Is sufficient information provided to allow interpretation of the expected output datasets and any results generated using the tool?

Yes

Reviewer Expertise:

cropping systems, sustainability, soil fertility, crop nutrition

I confirm that I have read this submission and believe that I have an appropriate level of expertise to confirm that it is of an acceptable scientific standard.
